# A clinically useful nomogram integrating bedside lung ultrasound and clinical parameters for pulmonary complications after non-thoracic surgery in blunt chest trauma patients

**DOI:** 10.3389/fmed.2026.1804376

**Published:** 2026-04-08

**Authors:** Xiaorui Han, Wen Chi, Peng Pang, Xiaobing Liu, Zhenguo Luo, Wenbo Cai, Li Zhang, Jianhong Hao

**Affiliations:** 1 Department of Anaesthesiology, HongHui Hospital, Xi'an JiaoTong University, Xi'an, Shaanxi, China; 2 Department of Operating Room, HongHui Hospital, Xi'an JiaoTong University, Xi'an, Shaanxi, China; 3 Department of Anaesthesiology, Binzhou Medical College Affiliated Hospital, Binzhou, Shaanxi, China

**Keywords:** chest injuries, fractures, postoperative complications, prediction model, ultrasonography

## Abstract

**Objectives:**

Lung ultrasonography can be used to effectively evaluate the severity of pulmonary injury. This study aimed to develop a nomogram that incorporated the results of lung ultrasonography and traditional clinical parameters to predict postoperative pulmonary complications (PPCs) in patients with blunt chest trauma who underwent non-thoracic surgery.

**Design:**

Prospective, observational study.

**Setting:**

The study was performed in a first 3A-grade hospitals in China.

**Participants:**

This study included 374 patients with blunt chest trauma who underwent extremity or pelvic fracture surgery.

**Interventions:**

All patients underwent lung ultrasonography before surgery and received general anesthesia combined with a regional block, goal-directed fluid therapy, and lung-protective ventilation. 30-days postoperatively, all participants were followed up to assess PPCs. Logistic regression was used to identify the key predictors of PPCs, and a nomogram was constructed. The predictive efficacy of the model was evaluated using receiver operating characteristic (ROC) and calibration curves, and the clinical application value was evaluated using decision curve analysis (DCA).

**Results:**

PPC incidence was 26.73%. American Society of Anesthesiologists physical status class, chronic obstructive pulmonary disease, recent respiratory infection, pneumothorax, lung ultrasonography score, and number of rib fractures were incorporated into the nomogram. The nomogram exhibited an excellent discriminative ability, with an area under the ROC curve of 0.932. The DCA demonstrated significant clinical utility of this nomogram in predicting PPCs.

**Conclusion:**

We developed a nomogram that combined the results of lung ultrasonography and traditional clinical parameters to predict the risk of PPCs in patients with blunt chest trauma who underwent non-thoracic surgery. This validation revealed satisfactory discrimination, indicating its potential clinical utility. This may assist in clinical decision-making.

## 1 Introduction

Traumatic fractures caused by traffic or motorcycle accidents and fall from a height are often associated with blunt chest trauma (1). Pulmonary contusion, the most frequently encountered pulmonary lesion after blunt chest trauma, is closely associated with postoperative pulmonary complications (PPCs) (2–5). PPCs can lead to prolonged postoperative tracheal intubation and increased costs and mortality (6). Therefore, the risk factors of PPCs should be identified in patients with blunt chest trauma, which will help in improving preventive and management strategies for PPCs.

Various models have been developed for predicting PPCs (7). Although these models are effective in most cases, their applicability in patients with blunt chest trauma is limited, as no indicators for assessing the severity of pulmonary injury are included in these models (8, 9). Lung ultrasonography can effectively evaluate the severity of pulmonary contusions caused by blunt chest trauma (10). In addition, it is repeatable, rapid, and non-invasive. Therefore, the development of a predictive model integrated with lung ultrasonography findings to stratify the risk of PPCs in patients with blunt chest trauma has a significant clinical value.

This study aimed to develop a nomogram that incorporated the results of lung ultrasonography and traditional clinical parameters to predict PPCs in patients with blunt chest trauma who underwent non-thoracic surgery.

## 2 Materials and methods

### 2.1 Ethics

The study protocol was approved by the Institutional Review Board. The trial is designed according to the principles of the Helsinki Declaration and is registered in the Chinese Clinical Trial Registry (ChiCTR2500102101). Written informed consent was obtained from all patients.

### 2.2 Sample size calculation

Sample size was calculated based on the Events Per Variable (EPV) metric, requiring a minimum of 10 positive events per independent variable to ensure model stability. Given the planned inclusion of nine predictor variables (age, American Society of Anesthesiologists [ASA] physical status class, chronic obstructive pulmonary disease [COPD], recent respiratory infection, preoperative hypoxemia, number of rib fractures, pleural effusion, pneumothorax, and lung ultrasonography score) were included, and the incidence of PPCs was 36.58% ^11^in patients with blunt chest trauma who underwent non-thoracic surgery.

The sample size was calculated as follows:


$$\begin{array}{l} \text{Required number of PPCs events}\geq \mathrm{EPV}\\ \times \text{Number of predictive variables}=10\times 10*=100 \end{array}$$

$$\text{Total sample size}\geq \frac{\text{Required number of PPCs events}}{\text{Predicted PPCs incidence}}=\frac{100}{0.3658}=273$$

*ASA physical status classification consists of three levels and was incorporated as two variables in the analysis.

### 2.3 Criteria for participation

Patients admitted with a diagnosis of blunt chest trauma who did not require thoracic surgical intervention and were scheduled to undergo elective surgery for extremity or pelvic fracture were included.

The diagnosis of blunt chest trauma was comprehensively established based on a clear history of blunt chest injury, combined with corresponding clinical manifestations and confirmatory imaging findings at admission. Specifically, the diagnostic criteria were defined as follows: patients must have a documented history of blunt force chest injury, including but not limited to traffic accidents, falls from height, crush injuries, and blunt impacts. Clinically, patients presented with at least one of the following manifestations: chest pain, chest wall tenderness, chest wall deformity, dyspnea, or decreased breath sounds on auscultation. In addition, imaging examinations (chest X-ray or chest computed tomography [CT]) confirmed at least one of the following pathological changes: rib fracture(s), sternal fracture, clavicular fracture, pulmonary contusion, pneumothorax, hemothorax, or chest wall soft-tissue injury.

The exclusion criteria were as follows: (1) head, facial, spinal, or abdominal injuries; (2) cardiac, liver, or kidney insufficiency; (3) perioperative cardiovascular and cerebrovascular accidents; and (4) undergoing unplanned surgery or discharged during follow-up; (5) refusal to participate.

### 2.4 Lung ultrasonography

**Figure 1:**
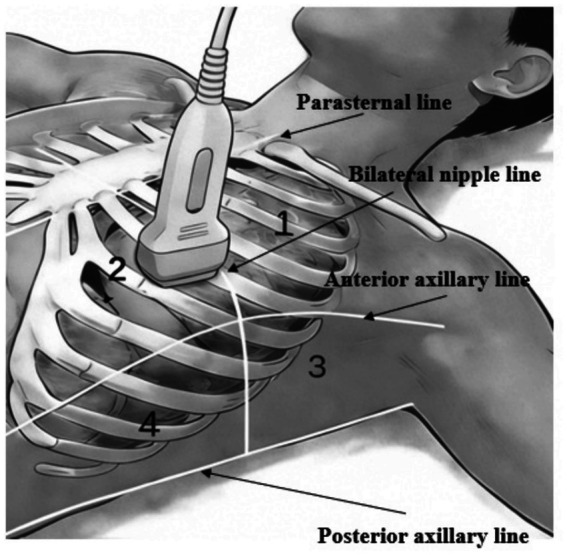
Regions in chest wall ultrasonographic examination. Based on the anatomical landmarks defined by the parasternal, anterior axillary, and posterior axillary lines, the hemithorax is divided into the anterior and lateral regions. Each region is further subdivided into superior and inferior subregions, bounded by the bilateral nipple line.

**Figure 2:**
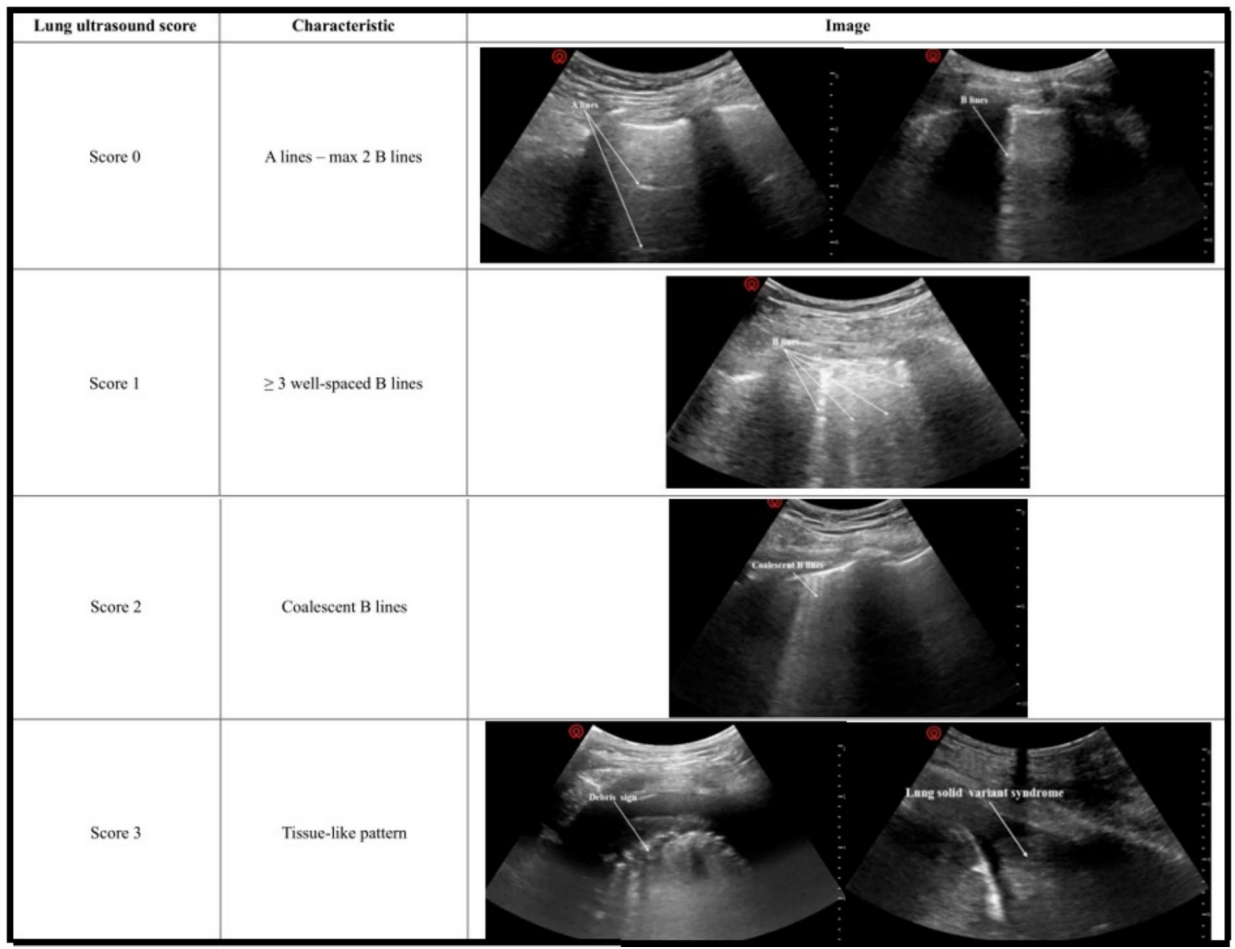
Lung ultrasound scoring criteria. 0 points for A-lines or ≤2 B-lines, 1 point for ≥3 well-spaced B-lines, 2 points for coalescent B-lines, and 3 points for tissue-like patterns (debris sign and lung solid variant syndrome). The final score for each region was the highest score observed on the ultrasound image.

Preoperative lung ultrasonography was performed by two ultrasonologists blinded to the study objectives using a convex transducer the day prior to surgery. Eight anatomical regions were defined on the chest wall (Figure 1), and a score was assigned to each region according to the criteria presented in Figure 2. The cumulative score of the eight regions was defined as the lung ultrasonography score (11, 12).

**Figure 3:**
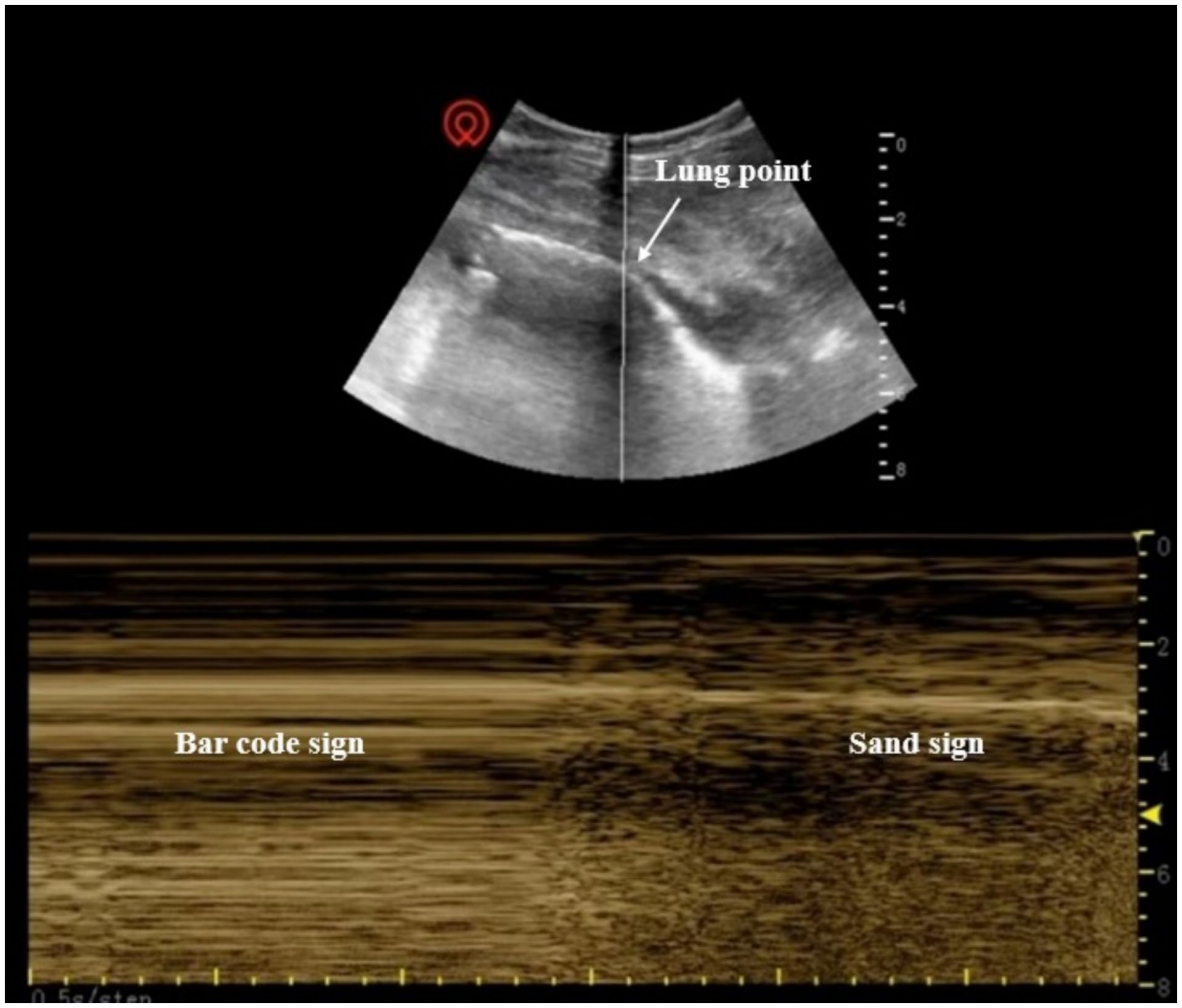
Pneumothorax on ultrasonography. Disappearance of the ultrasonographic “sand sign,” appearance of the “bar code sign,” or visible “lung point”.

**Figure 4:**
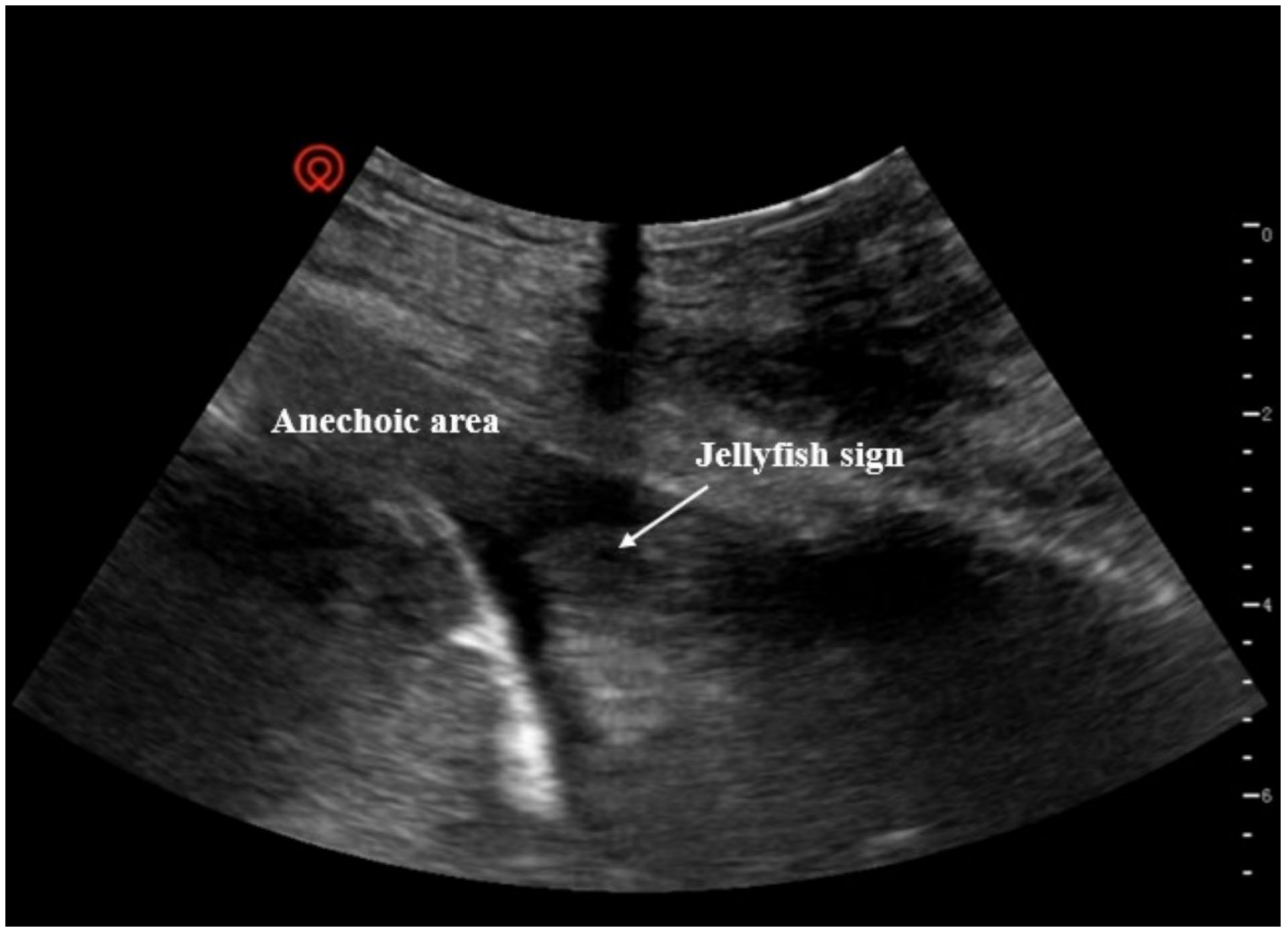
Pleural effusion on ultrasonography. Presence of anechoic areas in the pleural space, accompanied by signs of lung tissue compression.

In addition, the presence of pneumothorax (Figure 3) and pleural effusion (Figure 4) was determined based on the ultrasonographic findings (13). Closed thoracic drainage was performed before surgery in patients with pneumothorax or large- or moderate-volume pleural effusion accompanied by dyspnea, chest tightness, and peripheral oxygen saturation (SpO_2_) < 90% in room air. For patients who required preoperative chest tube placement, chest tubes were maintained until resolution of air leak and/or complete resolution of pleural effusion was confirmed by chest imaging or lung ultrasound. The indication and timing of chest tube removal were consistent across all patients.

### 2.5 Anesthesia protocol

General anesthesia combined with a regional block was administered, and the detailed protocol was as follows:

Induction: midazolam (0.1 mg·kg^−1^), sufentanil (0.25 μg·kg^−1^), and propofol (1.5–2.0 mg·kg^−1^)

Maintenance: remifentanil (0.1–0.2 μg·kg^−1^·min^−1^) and sevoflurane (1.0–1.5%); The bispectral index was maintained between 40 and 60;

Regional block: 10–20 mL of 0.33% ropivacaine solution

Neuromuscular blockade depth was monitored in all patients, and neostigmine was used to reverse the residual neuromuscular blockade.

### 2.6 Intraoperative ventilation strategies

Ventilation mode was set to volume-controlled ventilation, and the respiratory parameters were set as follows (14):

Tidal volume (TV): 6–8 mL·kg^−1^ of predicted body weight

Positive end-expiratory pressure (PEEP): 6–8 cm H₂O

Inspiratory to expiratory time ratio: 1:2

Respiratory rate: adequate to maintain an end-tidal carbon dioxide concentration of 35–40 mmHg

Fraction of inspired oxygen (FIO₂): <0.5

In case of SpO_2_ < 95%, rescue therapy, which consists of increasing FIO₂ and recruitment maneuvers, were initiated immediately.

### 2.7 Perioperative fluid and hemodynamic management

Before anesthesia induction, lactated Ringer’s solution was intravenously administered at a dose of 5 mL·kg^−1^, with subsequent continuous infusion maintained at 5 mL·kg^−1^·h^−1^. Under the monitoring of a continuous non-invasive arterial pressure system, 3 mL·kg^−1^ of lactated Ringer’s solution was intravenously infused every 15 min to maintain the pulse pressure variation (PPV) ≤ 14%. If the measured PPV persisted above 14% (duration ≥ 5 min) or the stroke volume (SV) increased by more than 10%, an additional 3 mL·kg^−1^ of lactated Ringer’s solution was administered until PPV ≤ 14% was achieved. If the PPV remained above 14% after fluid infusion, the aforementioned intervention was repeated. If, despite fluid resuscitation, the mean arterial pressure (MAP) was <60 mmHg, or if MAP < 60 mmHg was accompanied by PPV ≤ 14% and SV increase < 10%, vasopressor therapy was initiated: ephedrine 6 mg was first administered as an intravenous bolus, with a maximum cumulative dose not exceeding 30 mg; if necessary, norepinephrine was continuously infused at a rate of 0.05 μg·kg^−1^·min^−1^ (15).

### 2.8 Data collection

A data inventory was established based on previous clinical studies and systematic reviews, including the following patient-related risk factors: age, sex, height, weight, ASA physical status class, smoking status (within two months before surgery), COPD, recent respiratory infection (within one month before surgery), preoperative hypoxemia (SpO₂ ≤ 90% or partial pressure of arterial oxygen <60 mmHg on room air), and number of rib fractures; results of lung ultrasonography: pleural effusion, pneumothorax, and lung ultrasonography score; and procedure-related risk factors: perioperative blood transfusion volume, surgical site, duration of surgery and intravenous fluid volumes (7, 11, 16–18).

### 2.9 Follow up for PPCs

The first assessment of PPCs was performed between 6 p.m. and 10 p.m. postoperatively, followed by twice-daily evaluations (8 a.m. to 12 a.m. and 6 p.m. to 10 p.m.) until hospital discharge, with a maximum follow-up period of 30 days postoperatively. PPCs included respiratory infection, respiratory failure, bronchospasm, new-onset pneumothorax, and new-onset pleural effusion. Among them, new-onset pneumothorax was defined as pneumothorax identified on postoperative chest CT that was either absent preoperatively or showed a significant increase in extent compared with preoperative findings. New-onset postoperative pleural effusion was defined as a newly developed or significantly increased pleural effusion compared with the preoperative baseline, confirmed by postoperative chest CT. Other PPCs were defined according to the criteria outlined in the European Perioperative Clinical Outcome Guidelines (Supplementary File) (19).

### 2.10 Statistical analysis

Statistical analyses were conducted using the SPSS Software and R programming language. Categorical variables were analyzed using the chi-square test or Fisher’s exact test. Independent *t*-tests were used for continuous variables with a normal distribution, whereas the Mann–Whitney *U* test was used for variables without a normal distribution. Logistic regression was used to identify the key predictors of PPCs. Factors with a *p* < 0.05 in the univariate analysis were selected for multivariate logistic regression. A multicollinearity analysis was performed to assess the validity of the regression model. A nomogram prediction model was developed based on the results of multivariate logistic regression analysis. The area under the receiver-operating characteristic (ROC) curve (AUC) was calculated to evaluate the discriminative ability of the model. Internal validation was performed using the bootstrapping method with 1,000 resamples to correct for overfitting and evaluate the internal generalizability of the nomogram. The optimism-adjusted AUC was calculated to assess discriminative performance. The Hosmer-Lemeshow test and calibration curve (CC) were used to evaluate the goodness of fit of the model, and the decision curve analysis (DCA) was used to evaluate the clinical benefit of the model. Statistical significance was set at *p* < 0.05.

## 3 Results

**Figure 5:**
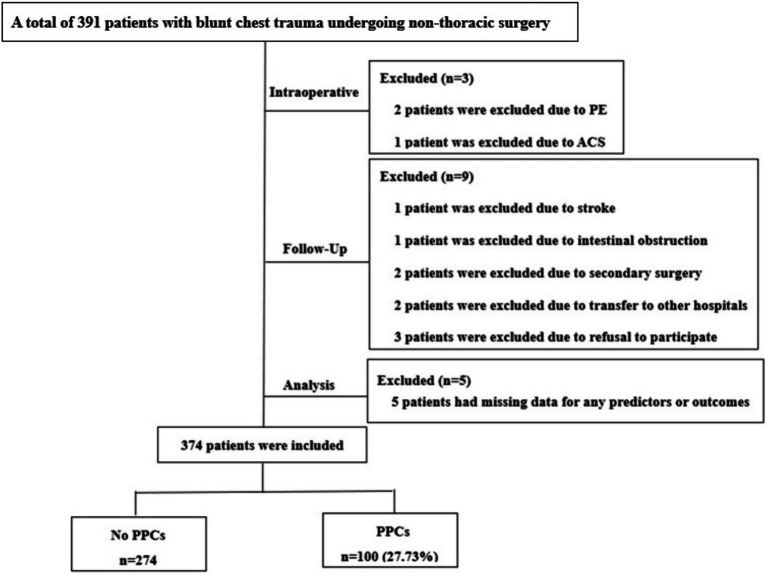
Study flowchart. PE, pulmonary embolism; ACS, acute coronary syndrome; PPCs, postoperative pulmonary complications.

A total of 374 patients who met the inclusion and exclusion criteria were analyzed between May 15, 2025 and December 5, 2025 (Figure 5).

### 3.1 Prevalence of PPCs and related variables

The incidence of PPCs was 26.73% (100/374). Specifically, 16.00% (16/100) patients presented with respiratory infection, 29.00% (29/100) with respiratory failure, 9.00% (9/100) with bronchospasm, 7.00% (7/100) with new-onset pneumothorax, 9.00% (9/100) with new-onset pleural effusion, and 30.00% (30/100) with multiple PPCs (two or more PPCs mentioned above). Postoperative length of hospital stay, unexpected ICU admission, and mortality increased significantly in patients with respiratory infection and multiple PPCs (Table 1).

**Table 1:** Postoperative length of hospital stay, ICU admission, and mortality according to the type of PPCs.

	No-PPCs	Respiratory infection	Respiratory failure	Pleural effusion	Pneumothorax	Bronchospasm	Multiple PPCs
Number	274	16	29	9	7	9	30
Postoperative length of hospital stay, M (Q₁, Q₃), days	6.0 (4.0, 10.0)	14.0 (9.0, 21.0)	10.0 (6.0, 14.0)	7.0 (4.0, 12.0)	7.0 (4.0, 11.0)	6.0 (3.0, 9.0)	17.0 (11.0, 22.0)
ICU admission, *n* (%)	6 (2.18%)	3 (18.75%)	3 (10.34%)	1 (11.11%)	2 (28.57%)	1 (11.11%)	7 (23.33%)
30-day mortality, *n* (%)	2 (0.72%)	1 (6.25%)	0	0	0	0	2 (6.66%)

Patients with and without PPCs differed significantly in terms of age, ASA physical status class, COPD, recent respiratory infection, preoperative hypoxemia, pleural effusion, pneumothorax, lung ultrasonography score, and number of rib fractures (*p* < 0.05) (Table 2).

**Table 2:** Demographic and clinical characteristics of the patients.

Variables	PPCs	non- PPCS	Overall	*p*
Number	100 (26.73%)	274	374	
Age, M (Q₁, Q₃), years	54.0 (35.0–68.0)	48.0 (33.0–62.0)	50.0 (34.0–63.0)	0.033*
**Sex**				**0.308**
Women	42	97	139	
Men	58	177	235	
**BMI (kg/m^2^)**				**0.229**
18.5–23.9	35	129	164	
<18.5	11	19	30	
24.0–27.9	44	100	144	
≥28.0	12	24	36	
**ASA classification**				0.001*
I	29	116	145	
II	41	121	162	
III	30	37	67	
Smoking status	20	58	78	0.852
COPD	30	22	52	0.000*
Recent respiratory infection	24	27	51	0.000*
Preoperative hypoxemia	18	19	37	0.001*
Pleural effusion	26	26	52	0.000*
Pneumothorax	20	33	53	0.045*
Lung ultrasound score, M (Q₁, Q₃)	8.0 (4.0, 10.0)	4.0 (3.0, 6.0)	5.0 (3.0–7.0)	0.000*
Number of rib fractures, M (Q₁, Q₃)	3.0 (2.0, 4.0)	1.0 (0.0, 1.0)	1.0 (1.0–2.0)	0.000*
**Operation type**				**0.552**
Pelvic surgery	26	77	103	
Extremity surgery	74	197	271	
Duration of surgical M (Q₁, Q₃), min	135.0 (100.0, 235.0)	163.0 (125.0, 208.0)	160.0 (120.0–210.0)	0.800
Intravenous fluid volumes, mL·kg^−1^·h^−1^	7.60 ± 0.90	7.56 ± 0.88	7.58 ± 0.88	0.890
Perioperative blood transfusion volume, U	2.0 (1.0, 3.0)	2.0 (0.0, 3.0)	2.0 (0.0–3.0)	0.437

PPCs, postoperative pulmonary complications; BMI, body mass index; ASA, American Society of Anesthesiologists; COPD, chronic obstructive pulmonary disease. *Significance at *p*-value < 0.05.

### 3.2 Logistic regression results

The results of univariate analysis showed that age (odds ratio [OR]: 1.014, 95% confidence interval [CI]: 1.001–1.027, *p* = 0.033), ASA physical status class (ASA physical status class III, OR: 3.243, 95% CI: 1.727–6.092, *p* = 0.000), COPD (OR: 5.000, 95% CI: 2.714–9.213, *p* = 0.000), recent respiratory infection (OR: 2.939, 95% CI: 1.601–5.396, *p* = 0.001), preoperative hypoxemia (OR: 2.994, 95% CI: 1.500–5.978, *p* = 0.002), pleural effusion (OR: 3.411, 95% CI: 1.866–6.234, *p* = 0.000), pneumothorax (OR: 1.857, 95% CI: 1.008–3.419, *p* = 0.047), lung ultrasonography score (OR: 1.945, 95% CI: 1.681–2.251, *p* = 0.000), and number of rib fractures (OR: 2.601, 95% CI: 2.097–3.225, *p* = 0.001) showed a significant association with PPC (Table 3).

**Table 3:** Univariate and multivariate logistic regression analysis for risk factors associated with PPCs.

Variables	Univariate		Multivariate	
	OR (95%CI)	*p*	AOR (95%CI)	*p*
Age (years)	1.014 (1.001–1.027)	0.033*	1.018 (0.997–1.040)	0.095
**Sex**				
Women	1.0 (reference)			
Men	0.783 (0.490–1.253)	0.308		
**BMI (kg/m^2^)**				
18.5–23.9	1.0 (reference)			
<18.5	1.340 (0.550–3.268)	0.520		
24.0–27.9	1.622 (0.969–2.714)	0.066		
≥28.0	1.843 (0.839–4.049)	0.128		
**ASA classification**				
I	1.0 (reference)		1.0 (reference)	
II	1.311 (0.763–2.254)	0.326	1.327 (0.565–3.119)	0.516
III	3.243 (1.727–6.092)	0.000*	3.133 (1.221–8.036)	0.017*
Smoking status	1.056 (0.597–1.866)	0.852		
COPD	5.000 (2.714–9.213)	0.000*	3.089 (1.078–8.853)	0.036*
Recent respiratory infection	2.939 (1.601–5.396)	0.001*	3.535 (1.338–9.342)	0.011*
Preoperative hypoxemia	2.994 (1.500–5.978)	0.002*	2.228 (0.739–6.716)	0.155
Pleural effusion	3.411 (1.866–6.234)	0.000*	1.756 (0.563–5.480)	0.332
Pneumothorax	1.857 (1.008–3.419)	0.047*	3.265 (1.317–8.092)	0.011*
Lung ultrasound score	1.945 (1.681–2.251)	0.000*	1.907 (1.561–2.329)	0.000*
Number of rib fractures	2.601 (2.097–3.225)	0.000*	2.403 (1.823–3.168)	0.000*
**Operation type**				
Pelvic surgery	1.0 (reference)			
Extremity surgery	1.172 (0.694–1.979)	0.553		
Duration of surgical	1.001 (0.998–1.003)	0.720		
Intravenous fluid volumes	0.957 (0.521–1.759)	0.887		
Perioperative blood transfusion volume	1.201 (0.874–1.547)	0.437		

OR: odds ratio; AOR, adjusted odds ratio; CI, confidence interval; PPCs, postoperative pulmonary complications; BMI, body mass index; ASA, American Society of Anesthesiologists; COPD, chronic obstructive pulmonary disease. *Significance at *p*-value < 0.05.

The results of multivariate analysis showed that ASA physical status class III (OR: 3.133, 95% CI: 1.221–8.036, *p* = 0.017) and combined COPD (OR: 3.089, 95% CI: 1.078–8.853, *p* = 0.036), recent respiratory infection (OR: 3.535, 95% CI: 1.338–9.342, *p* = 0.011), and pneumothorax (OR: 3.265, 95% CI: 1.317–8.092, *p* = 0.011) were independent risk factors for PPCs. A higher lung ultrasonography score (OR: 1.907, 95% CI: 1.561–2.329, *p* = 0.000) and a greater number of rib fractures (OR: 2.403, 95% CI: 1.823–3.168, *p* = 0.000) were significantly associated with an increased risk of PPCs (Table 3).

### 3.3 Model collinearity diagnostics

As shown in the Table 4, all variables included in the multivariable analysis had Variance Inflation Factor (VIF) values less than 5, and tolerance levels greater than 0.1, thereby ruling out the issue of multicollinearity among the independent variables.

**Table 4:** Model collinearity diagnostics.

Variables	VIF	Tolerance
Age (years)	1.18	0.84
ASA classification	1.07	0.92
COPD	1.17	0.85
Recent respiratory infection	1.06	0.94
Preoperative hypoxemia	1.19	0.83
Pleural effusion	1.33	0.75
Pneumothorax	1.01	0.98
Lung ultrasound score	1.45	0.68
Number of rib fractures	1.30	0.76

VIF: variance inflation factor.

### 3.4 Construction and validation of the nomogram model

**Figure 6:**
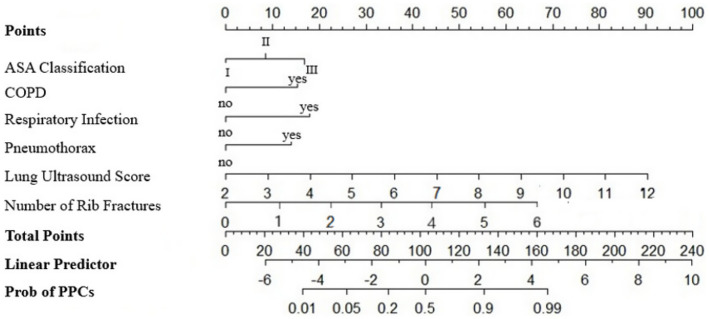
Nomogram for predicting the risk of pulmonary complications after non-thoracic surgery in patients with blunt thoracic trauma. To use the nomogram, a vertical line is drawn to the top point row to assign points for each variable. Then, the total number of points is calculated, and a vertical line is drawn downward from the total point row to determine the probability of PPCs.

**Figure 7:**
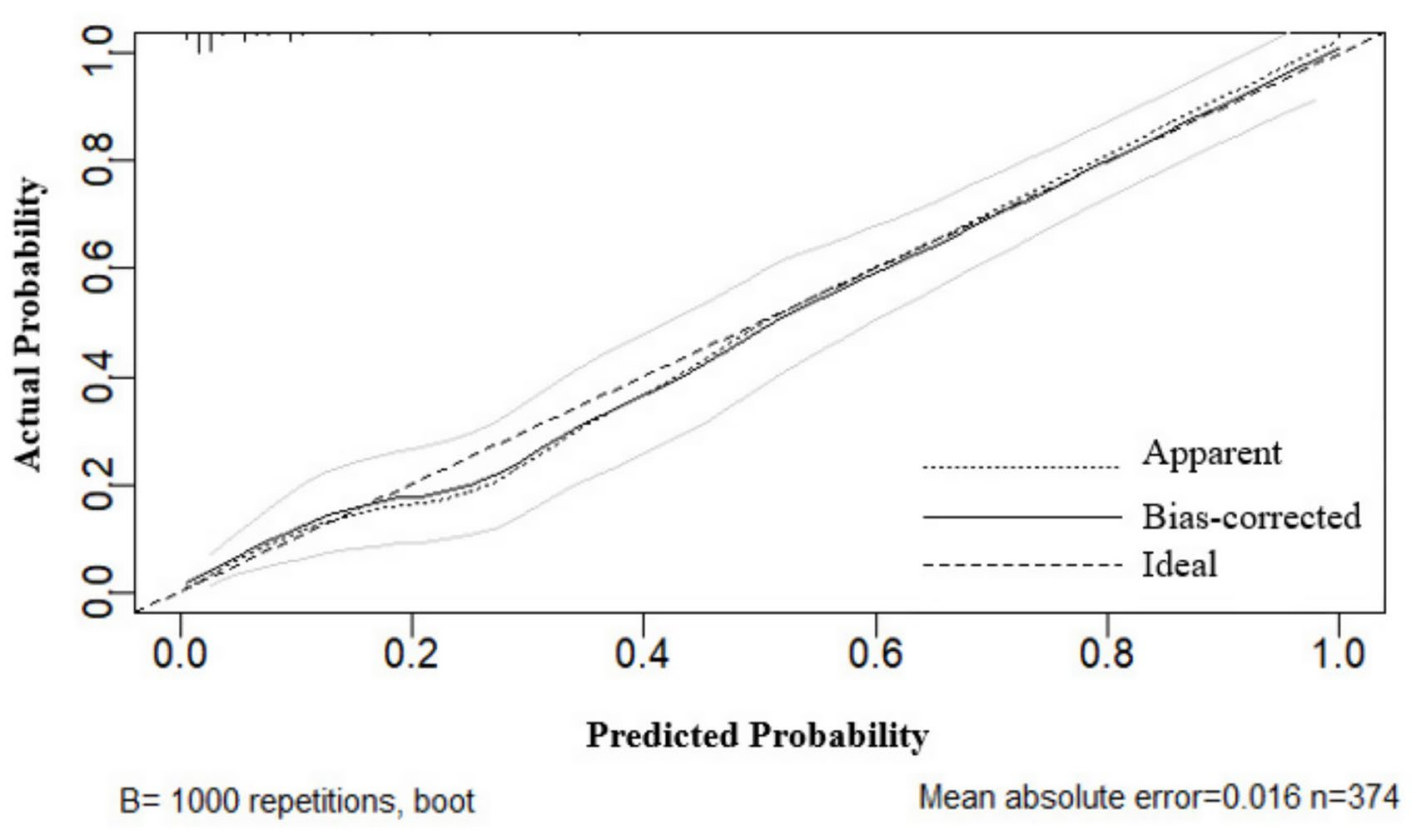
The calibration and ideal prediction curves in agreement.

**Figure 8:**
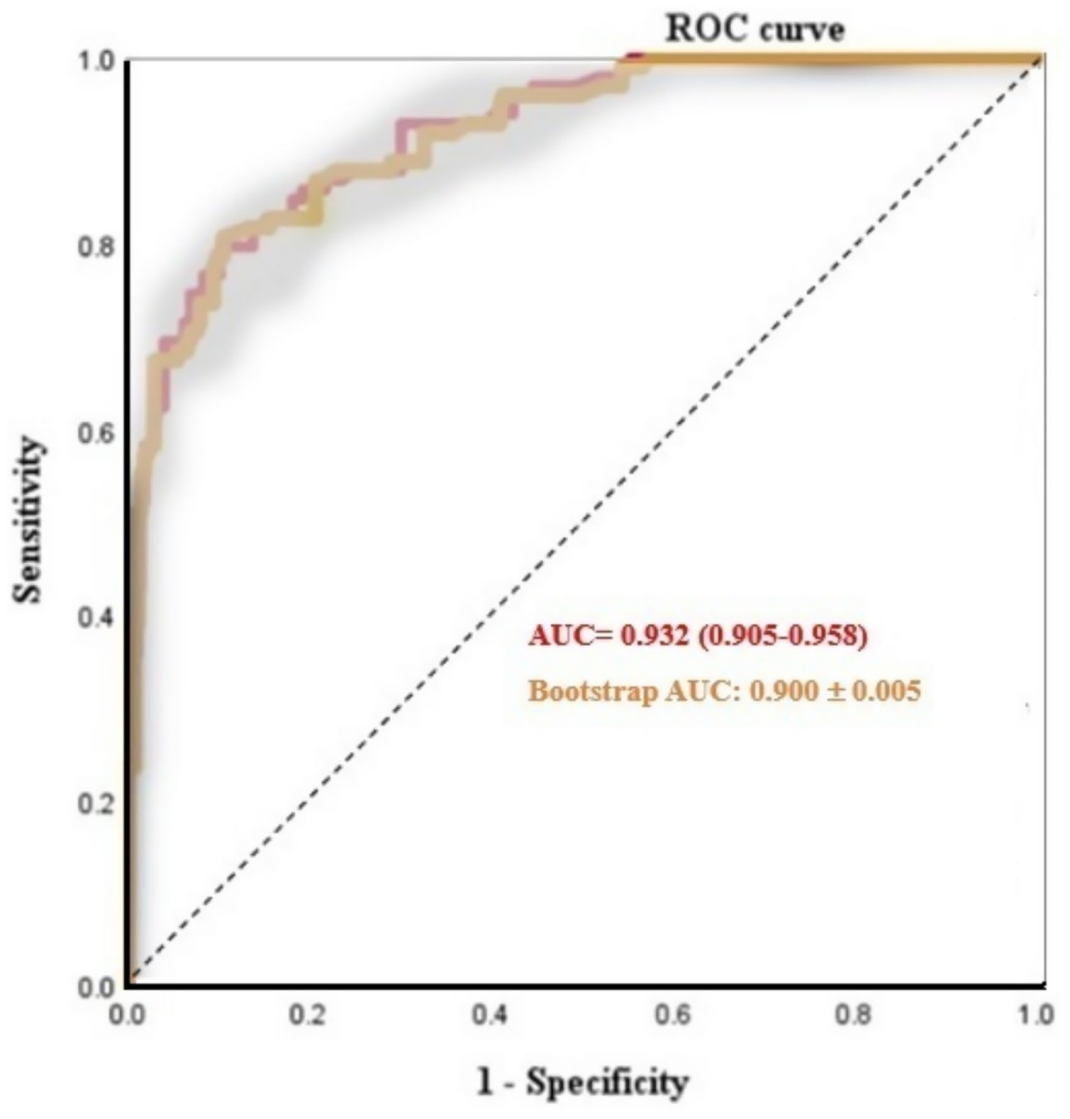
Receiver operating characteristic curve of the model on the original data and bootstraps validation.

**Figure 9:**
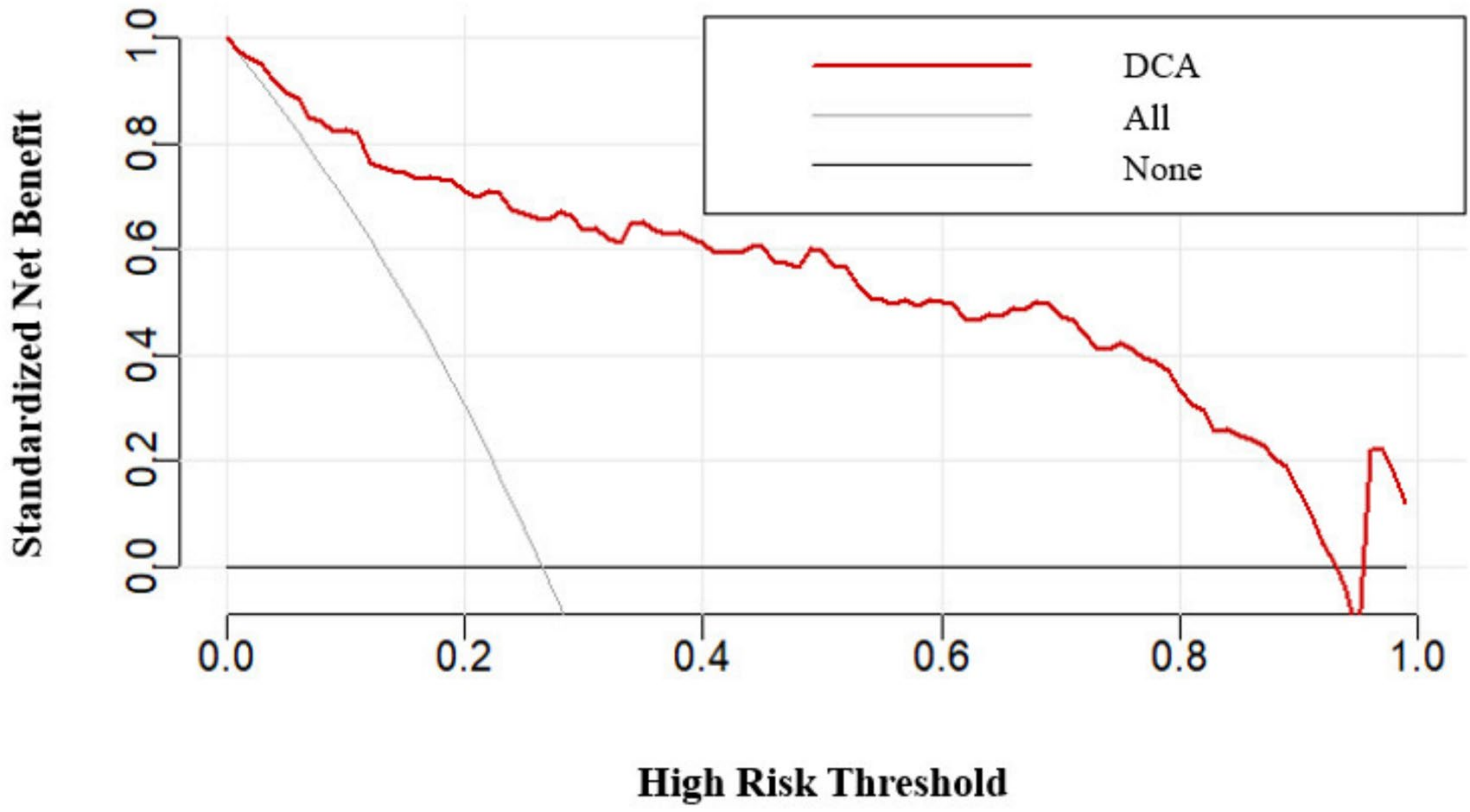
Decision curve analysis of the prediction nomogram.

Following the identification of independent risk factors using logistic regression, a predictive nomogram was successfully developed (Figure 6). The Hosmer-Leme-show test yielded a *χ*^2^ value of 5.16 (*p* = 0.740). The CC showed a good consistency with the ideal prediction curve, with a mean absolute error of 0.016 (Figure 7). The ROC curves demonstrated that the nomogram exhibited a higher AUC (AUC = 0.932, 95% CI: 0.905–0.958), and the bootstrap-corrected AUC was highly consistent with the original AUC, suggesting minimal overfitting and good generalizability of the nomogram (Figure 8). DCA results confirmed that the model possessed a good clinical predictive value and applicability (Figure 9). To enhance the convenience and practicability of the nomogram, we developed web-based calculators^1^ to predict the risk of developing PPCs.

## 4 Discussion

### 4.1 Incidence of PPCs

The incidence of PPCs was 26.73% (100/374), lower than the 36.58 and 30.90% reported in previous studies (11, 20). By examining the underlying causes, we identified two key factors that contributed to this issue: intraoperative fluid management and intraoperative ventilation strategy, both of which are closely associated with the occurrence of PPCs (21, 22). In this study, for all patients, goal-directed fluid therapy, and lung-protective ventilation strategies were adopted.

In thoracic or non-thoracic surgery, restrictive fluid replacement or goal-directed fluid therapy can reduce the incidence of PPCs and reduce the postoperative hospital stay of patients (23, 24). During thoracic trauma, alveolar capillary injury occurs, leading to increased vascular permeability (25). Pulmonary edema is more likely to occur in the context of volume overload (26). Therefore, in patients with blunt chest trauma, restrictive fluid replacement or goal-directed fluid therapy is of greater importance.

Mechanical ventilation following pulmonary contusion induces local and systemic inflammation (27). Therefore, a mechanical ventilation strategy for patients with concurrent pulmonary contusion is a significant clinical challenge. A low TV exerts a protective effect against PPCs in patients, regardless of concurrent lung injury (28–30). However, the optimal level of PEEP and lung recruitment maneuvers is controversial. High PEEP levels and lung recruitment maneuvers can lead to hemodynamic impairment (31, 32). Hong et al. (33) observed that high PEEP significantly increases the levels of bronchiolar inflammatory markers, suggesting that it can induce lung injury. Therefore, a moderate PEEP (6–8 cm H₂O) was selected in this study, and according to the recommendations of Guldner et al. (34), lung recruitment maneuvers were only performed for desaturation. A combination of low TV and moderate PEEP effectively prevents the occurrence of PPCs (14, 35, 36).

In addition, residual neuromuscular blockade is associated with PPCs (36–38). McLean et.al (39) found that PPCs were associated with the dosage of neuromuscular blocking agents, and the strength of this association could be attenuated by reversal of the neuromuscular blockade. In this study, all patients were monitored for the depth of neuromuscular blockade during anesthesia, and residual neuromuscular blockade was antagonized.

In summary, for patients with blunt thoracic trauma, goal-directed fluid therapy, ventilation strategies with a low TV, moderate PEEP, routine neuromuscular blockade monitoring, and timely neuromuscular blockade reversal should be implemented during the perioperative period. These measures can reduce the incidence of PPCs.

### 4.2 Risk factors of PPCs

Age, obesity, ASA physical status class, smoking history, COPD, respiratory infection, and hypoxemia are risk factors for PPCs (7, 16, 17). In this study, in addition to these risk factors identified in previous studies, the predictive model incorporated lung ultrasonography results (pleural effusion, pneumothorax, and lung ultrasonography score) and number of rib fractures. Finally, six variables were included in the nomogram for predicting PPCs: ASA physical status class, COPD, respiratory infection, pneumothorax, lung ultrasonography score, and number of rib fractures.

#### 4.2.1 ASA physical status class

The ASA classification is a commonly used patient risk assessment standard in anesthesiology departments. It was initially designed to predict perioperative mortality, but it can also predict PPCs (40). Patients with an ASA physical status class > II have a significantly increased risk of PPCs (41–44). Further, in patients with trauma, ASA physical status class is a statistically significant predictor of postoperative mortality (45, 46). Therefore, patients with blunt chest trauma and ASA physical status class > II should be monitored carefully. Active preoperative optimization of patients’ overall physical status may effectively reduce the risk of postoperative mortality, thereby improving clinical treatment outcomes and patient prognosis.

#### 4.2.2 COPD

COPD is closely associated with PPCs (47). The impact of COPD on PPCs is more prominent in patients with blunt chest trauma. Blum et al. (48) found that in patients with blunt chest trauma, COPD is an important risk factor for postoperative acute respiratory distress syndrome. Avcı et al. (49) found that pneumonia, prolonged mechanical ventilation, sepsis, and prolonged intensive care unit and total hospital stays were common in patients with COPD after chest trauma. Shoko et al. (50) also showed a significant increase in mortality in patients with trauma and COPD. Lee et al. (51) suggest that, the overall condition of patients with COPD, such as presence of diminished breath sounds or wheezing, should be controlled before surgery. If acute exacerbation of symptoms or stridor occurs, the patient should be treated with bronchodilators and may require systemic corticosteroid therapy before surgery (51).

#### 4.2.3 Respiratory infection

Recent respiratory infections are strong PPCs risk factors (52, 53). They can induce airway hyper-reactivity, decreased pulmonary function, and impaired immune function (9). This is particularly true in patients with trauma, as traumatic injury disrupts immune system homeostasis, rendering them more susceptible to infections (54, 55). Given the persistence of airway inflammation even after the resolution of infectious pathogens, Lumb et al. (56) suggest that for patients with respiratory infections or inadequately treated respiratory diseases, surgery should be performed only after their conditions have been fully treated. However, this approach may not be appropriate for patients with trauma. Hall et al. (57) confirmed that nebulized lidocaine can reduce airway hyperreactivity induced by upper respiratory tract infection. In addition, they also found that recent respiratory tract infections can lead to excessive tracheal secretions, which may be associated with postoperative pneumonia (57). Prophylactic mucolytics may be beneficial in patients with a recent history of respiratory infection. Ambroxol can reduce the viscosity of bronchial sputum, thereby helping in expectorating sputum (58, 59).

#### 4.2.4 Pneumothorax

The incidence of occult pneumothorax in patients with blunt chest trauma is 2–55% (60). Bokhari (61) and Ho et al. (62) suggest that in patients with blunt thoracic trauma, chest radiography or computed tomography should be the preferred initial imaging modality. However, ultrasonography is radiation-free and has a high repeatability and rapid operation. It is widely applied in lung examinations, and has a high diagnostic accuracy for pneumothorax and pleural effusion (63). In this study, we diagnosed pneumothorax and pleural effusion based on ultrasonographic findings. Richter et al. (64) suggest that patients receiving mechanical ventilation should undergo tube thoracostomy to prevent tension pneumothorax. We found that pneumothorax, which may be associated with ventilator-associated lung injuries, remained an independent risk factor for PPCs in patients with blunt thoracic trauma complicated by pneumothorax, even when closed thoracic drainage was performed before surgery. Richter et al. (64) suggest that in patients with blunt thoracic trauma complicated by pneumothorax, if mechanical ventilation is required, the peak, plateau, and end-expiratory pressures and tidal volume should be limited. In addition, for patients with pneumothorax, the selection of neuraxial anesthesia or nerve block anesthesia may be beneficial.

#### 4.2.5 Lung ultrasonograhic scores

During pulmonary contusion, the pulmonary barrier function undergoes progressive impairment, which subsequently leads to changes in the air-to-fluid ratio of the lung tissue (26). Ultrasonography can assess the severity of pulmonary contusion based on this change (65). In this study, we quantified the severity of lung injury using the lung ultrasonographic score and found that a higher lung ultrasonographic score was significantly correlated with an elevated risk of PPCs. The predictive value of lung ultrasonography for postoperative respiratory function has been reported. He et al. (22) reported that lung ultrasonography was efficient in predicting pulmonary insufficiency after thoracic surgery. Goel et al. (66) further quantified this technique and found that a lung ultrasonography score >10 could serve as an effective predictive threshold for the need for postoperative oxygen therapy. In addition, Dransart-Raye et al. (67) showed an association between a high lung ultrasonography score and low oxygenation index in postoperative intensive care unit patients. Therefore, for patients with a high lung ultrasound score, preoperative interventions and the selection of optimal surgical timing based on ultrasound score results may yield clinical benefits.

#### 4.2.6 Number of rib fractures

Rib fracture is one of the most common type of injury in thoracic trauma (68). The number of rib fractures is associated with the severity of thoracic trauma and the risk of severe complications and mortality (69–71). In this study, we found that an increased PPC risk was significantly associated with a greater number of rib fractures. In addition, the fracture site and the presence of displacement are important factors affecting pulmonary complications. Chien et al. (18) found that patients with rib fractures on both sides or displaced rib fractures had a higher incidence of pulmonary complications. Livingston et al. (72) reported that patients with rib fractures distributed across multiple anatomical regions have an increased incidence of complications. Preoperative pulmonary function exercises and active pain management can reduce the incidence PPCs. Sum et al. (73) found that incentive spirometry reduced the incidence of pulmonary complications in patients with rib fractures. Ekpe et al. (74) found that adequate analgesia can reverse the adverse effects of chest pain caused by multiple traumatic rib fractures on pulmonary function parameters by improving respiratory function.

In this study, the univariate analysis showed that PPCs were associated with age, preoperative hypoxemia, and pleural effusion. However, the multivariate analysis indicated that these factors were not independent risk factors for PPCs. This may be because older patients and those with hypoxemia are more likely to have a higher ASA physical status class. In patients with pleural effusion, compression of the surrounding lung tissue by the effusion results in reduced pulmonary ventilation, leading to a higher lung ultrasonographic score.

Notably, we found that BMI and smoking status were not risk factors for PPCs. Obesity is an independent risk factor for PPCs, but the studies reporting this result were performed in patients undergoing thoracic or major abdominal surgery (75, 76). In orthopedic surgery, numerous studies have confirmed that the occurrence of PPCs was not associated with BMI (77, 78). Currently, the association between smoking and PPCs is conflicting (9, 79–81). Smoking is identified as a risk factor for PPCs and patients can benefit from quitting smoking only if they quit for at least 2–4 weeks before surgery (82). However, surgery in patients with trauma should not be delayed. Therefore, for patients who smoke, the impact of smoking on cardiopulmonary function should be carefully assessed and intensive perioperative care and monitoring should be provided.

### 4.3 Limitations

This study had three limitations. First, it was a single-center study. Therefore, the nomogram should be used in combination with sound clinical judgment, individual patient conditions, and available medical resources. Future research should consider conducting multicenter studies with larger sample sizes to further validate the reliability and universality of the conclusions drawn in this study. Second, not all factors influencing PPCs, especially hypoalbuminemia and anemia, known risk factors (83–85), were analyzed in this study. In this study, preoperative corrective treatment was administered to patients with a serum albumin level <30 g·L^−1^ and hemoglobin concentration <10 g·dL^−1^. This may be another important reason for the low incidence of PPCs in this study. Manosroi et al. (86) found that in patients with hip fractures and moderate to severe anemia at admission, increasing the preoperative hemoglobin level to ≥10 g·dl-1 can significantly reduce the risk of postoperative complications and mortality. Third, this study excluded patients with head and neck, spinal, and abdominal injuries and those with abnormal hepatic or renal function indicators. Further research is required for this cohort of patients.

## 5 Conclusion

This study analyzed the lung ultrasonography images and traditional clinical parameters in patients with blunt thoracic trauma. Logistic regression analysis identified six predictive factors for PPCs: ASA physical status class, COPD, recent respiratory infection, pneumothorax, lung ultrasonographic score, and number of rib fractures. Based on these factors, a nomogram prediction model was constructed. Internal validation results confirmed that the nomogram performed excellently in predicting the onset of PPCs. However, since it has not undergone large-sample external validation, the present nomogram is intended to serve as an adjunctive supportive tool for clinical judgment rather than a decisive basis. Its clinical value should be interpreted in the context of the patient’s specific clinical situation.

## Data Availability

The raw data supporting the conclusions of this article will be made available by the authors, without undue reservation.

## Footnotes

1
https://haojianhong722.shinyapps.io/dynnomapp
