# Supplementary material for: A clinically useful nomogram integrating bedside lung ultrasound and clinical parameters for pulmonary complications after non-thoracic surgery in blunt chest trauma patients
Source: Front Med (Lausanne). 2026 Apr 8;13:1804376. doi: 10.3389/fmed.2026.1804376 (PMC13099831; doi:10.3389/fmed.2026.1804376)
Supplement: Supplementary file 1 [file Table_1.docx]

| **Definitions of postoperative pulmonary complications** | |
| --- | --- |
| Complication | Definition |
| Respiratory infection | Patient has received antibiotics for a suspected respiratory infection and met one or more of the following criteria: new or changed sputum, new or changed lung opacities, fever, white blood cell count >12x10^9^/L. |
| Respiratory failure | Postoperative PaO_2_<8kPa(60mmHg) on room air, a PaO_2_:F0_2_ ratio<40kPa (300mmHg) or arterial oxyhaemoglobin saturation measured with pulse oximetry < 90% and requiring oxygen therapy. |
| Pleural effusion | Chest radiograph demonstrating blunting of the costophrenic angle, loss of sharp silhouette of the ipsilateral hemidiaphragm in upright position evidence of displacement of adjacent anatomical structures or (in supine position) a hazy opacity in one hemithorax with preserved vascular shadows. |
| Atelectasis | Lung opacification with a shift of the mediastinum, hilum or hemidiaphragm toward the affected area, and compensatory over-inflation in the adjacent non-atelectatic lung. |
| Pneumothorax | Air in the pleural space with no vascular bed surrounding the visceral pleura. |
| Bronchospasm | Newly detected expiratory wheezing treated with bronchodilators. |
| Aspiration pneumonitis | Acute lung injury after the inhalation of regurgitated gastric contents. |
